# Significant Promising Effects of Bariatric Surgery on the Biochemical Control of Glycemia and Lipidemia in Diabetic Patients in Western Saudi Arabia: A Tertiary Center Experience and a Retrospective Study

**DOI:** 10.7759/cureus.53295

**Published:** 2024-01-31

**Authors:** Ibrahim Abdel-Rahman, Abdulhamid Awadh Alharbi, Maryam Zain Alsaedi, Noof Mejzi Alamri Alharbi, Sajidah Basheer Al-Mughassil, Zainab Anwar Al-Bahar, Abdel-Raheem Donkol, Hussam Baghdadi, Mariam Eid Alanzi, Salah Mohamed El Sayed

**Affiliations:** 1 Department of Surgery, Faculty of Medicine, Al-Rayyan Medical Colleges, Al-Madinah Al-Munawwarah, SAU; 2 Department of General Surgery, Obesity Center, King Fahad General Hospital, Al-Madinah Al-Munawwarah, SAU; 3 College of Medicine, Faculty of Medicine, Al-Rayyan Medical Colleges, Al-Madinah Al-Munawwarah, SAU; 4 Department of General Surgery, Faculty of Medicine, Aswan University, Aswan, EGY; 5 Department of Clinical Biochemistry and Molecular Medicine, Faculty of Medicine, Taibah University, Al-Madinah Al-Munawwarah, SAU; 6 Division of Diabetology, Diabetes Center, Al-Madinah General Hospital, King Salman Bin Abdul-Aziz Medical City, Al-Madinah Health Cluster, Al-Madinah Al-Munawwarah, SAU; 7 Department of Clinical Biochemistry, Faculty of Medicine, Sohag University, Sohag, EGY

**Keywords:** bmi, bariatric surgery, lipidemic control, glycemic control, diabetes mellitus

## Abstract

Background: The prevalence of obesity has increased globally and is associated with many comorbidities such as type 2 diabetes and fatty liver and cardiovascular diseases. Bariatric surgery is considered an effective intervention for achieving weight loss and controlling lipidemia and glycemia.

Objectives: This Saudi retrospective observational study evaluates the clinical and biochemical benefits following bariatric surgery to obese diabetic patients.

Methodology: After gaining ethical committee approval, data was collected from the patients' medical records at a tertiary medical center (King Fahad General Hospital, Al-Madinah Al-Munawwarah, Saudi Arabia). The total sample size was 61 patients, of whom 78.33% (n=48) had a body mass index (BMI) of 40 or greater (obese class III).

Results: Following bariatric surgery, there were statistically significant reductions (p<0.001) in BMI and HbA1C (decreased from 45.53±7.791 kg/m^2 ^and 7.9±1.82% to 33.42±6.18 kg/m^2 ^and 6.06±1.35%, respectively, after surgery). Likewise, significant reductions (p<0.001) occurred to serum total cholesterol, low-density lipoprotein (LDL) cholesterol, and triglycerides that decreased from 234.4±26.7 mg/dl, 152.2±19.4 mg/dl, and 187.3±24.6 mg/dl to 158.4±17.3 mg/dl, 95.6±15.7 mg/dl, and 132.5±19.5 mg/dl, respectively. Interestingly, serum high-density lipoprotein (HDL) significantly increased (p<0.001) from 43.8±6.2 mg/dl to 52.3±4.6 mg/dl. Using the novel clinical therapeutic index, bariatric surgery decreased BMI by about 26.6%. Using the novel biochemical therapeutic index, bariatric surgery decreased HbA1C, serum total cholesterol, serum LDL cholesterol, and serum triglycerides by about 22.99%, 32.42%, 37.18%, and 29.26%, respectively, while serum HDL increased by about 19.4%.

Conclusion: Bariatric surgery is an effective intervention for obese diabetic patients resulting in weight loss, better control of diabetes and hyperlipidemia, and the metabolic profile. It is also recommended in Saudi Arabia for the high prevalence of obesity and diabetes mellitus.

## Introduction

Obesity is a globally epidemic disease that affects around 650 million people worldwide, and it is proven that longstanding obesity increases the risk of hepatic, metabolic, and cardiovascular morbidities and related mortality in addition to physical and psychological complications, including type 2 diabetes mellitus [1]. As type 2 diabetes and obesity are closely linked, patients with both diseases present a challenge to their clinicians [2]. Type 2 diabetes and liver disease are strongly correlated with obesity, and liver disease caused by obesity is a frequent reason for liver transplantation. In Saudi Arabia, the prevalence of obesity has risen in recent years. Compared to the global average of 11% for males and 15% for women in 2016, the national statistics for Saudi Arabia indicate that in 2013, 24.1% of Saudi men and 33.5% of Saudi women were obese. Type 2 diabetes is correspondingly prevalent and on the rise in Saudi Arabia; the risk factor for this condition is increased by more than seven times due to obesity. Additionally, non-alcoholic fatty liver disease (NAFLD), which affects over 30% of Middle Easterners and may affect 48% of Saudi Arabians by 2030, is also linked to obesity. Non-alcoholic steatohepatitis (NASH), a more severe form of NAFLD, has been connected to advanced liver fibrosis, cirrhosis, and liver cancer. In Saudi Arabia, NASH is now the most common reason for liver transplants and exceeds the hepatitis C virus. The financial and health costs of type 2 diabetes, liver disease, and liver cancer could be dramatically reduced with effective obesity control policies and lifestyle interventions [3].

In 2021, the global prevalence of diabetes is estimated to be 10.5% among people aged 21-80, with a suspected increase to 12.2% by 2045 [4]. Saudi Arabia has one of the highest rates of chronic diseases globally and is the highest in the Arabian Gulf region; the prevalence of diabetes was found to be 28% between 2016 and 2022 [5]. Epidemiological data indicate that chronic diseases are responsible for 70% of all deaths in Saudi Arabia [6].

Long-term management through anti-hyperglycemic medications and statins, together with lifestyle modifications, is essential to prevent diabetic complications and decrease the development of diabetes-associated sequelae [7]. This point was illustrated by a study conducted among the 50 largest metropolitan areas in the USA that reported that more than 44% of insured diabetic patients who received anti-hyperglycemic drugs were considered to be poorly controlled. In addition, patients with diabetes and obesity had more difficulties maintaining weight loss [8]. Indeed, there is a need for different therapeutic approaches in addition to the traditional tools. Bariatric surgery was found to be more effective in the management of obesity and can promote diabetic remission for patients with difficulty controlling their glycemic level. Bariatric surgery was found to be beneficial to glucose homeostasis through decreasing insulin resistance and increasing insulin secretion [9].

A meta-analysis study revealed that bariatric surgeries were associated with greater weight loss, a better lipid profile, increased remission rates of DM, and improved quality of life when compared with non-surgical management [10]. However, despite these good results, other studies reported that only 15% of treated surgical diabetic patients maintained remission for 10 years [11].

Interestingly, studies reporting the impact of bariatric surgeries on glucose hemostasis are still more extensive and need to be elucidated. Therefore, this study was conducted to explore the association between bariatric surgery and glycemic control among patients with diabetes mellitus.

## Materials and methods

Methodology

Ethical Committee Approval

Ethical approval was obtained from the Institutional Review Board (IRB) of the Faculty of Medicine, Al-Rayyan Medical Colleges, Al-Madinah Al-Munawwarah, Saudi Arabia (approval number: HA-03-M-122-008), according to the National Committee of Bioethics (NCBE). Prior to the study, the issue of confirming anonymity was addressed to participants. Confidentiality and privacy were maintained by encoding data and eliminating personally identifiable information.

A Retrospective Study

This retrospective observational study was conducted at King Fahad General Hospital in Al-Madinah Al-Munawwarah, Saudi Arabia. This study aimed at evaluating the clinical and biochemical effects of bariatric surgeries on patients with obesity and type 2 diabetes mellitus for achieving many objectives, e.g., to determine the improvement in body mass index (BMI) of the obese diabetic patients who underwent bariatric surgery and to investigate the association between bariatric surgery and both glycemic and lipidemic control among obese patients with diabetes mellitus. This study also aimed at assessing the changes in glycated hemoglobin among obese diabetic patients who have undergone bariatric surgery.

Data was collected from the patients' medical records at King Fahad General Hospital. Inclusion criteria were all of the following: patients >18 years old who were diagnosed with type 2 diabetes mellitus, having an HbA1C of >6.5% and a BMI of >30 (considered to be obese), and already underwent bariatric surgeries in the previous year. Exclusion criteria were any of the following: those having chronic cardiac disorders, older than 50 years, or having concurrent or recent participation in other studies.

Sample Size

The sample size included the patients who met the inclusion criteria with available clinical data. The sample was calculated, with a total of 61 patients (based also on data availability in light of the inclusion and exclusion criteria). We collected the data from the patients' medical records, including basic demographic characteristics such as age, gender, employment, and social status; BMI before and after surgery; past medical history and duration of diabetes mellitus; and laboratory investigations such as HbA1C before and after surgery.

Clinical Therapeutic Index (CTI)

It is a modification of our novel previously reported "clinical therapeutic index" [12]. CTI is the percentage improvement of a studied clinical parameter (such as BMI) following a medical procedure such as Al-hijamah (prophetic wet cupping therapy) or bariatric surgery. It is calculated using the following formula: CTI=(100×(the clinical parameter prior to bariatric surgery−same clinical parameter after surgery)/the clinical parameter prior to bariatric surgery). CTI is quite helpful to judge the surgical benefits and outcomes.

Biochemical Therapeutic Index (BTI)

It is also a modification of our novel previously reported "clinical therapeutic index" [12]. The novel BTI is the percentage reduction of an investigated biochemical parameter (such as serum total cholesterol, triglycerides, low-density lipoprotein (LDL), and others) or the percentage increase of a useful biochemical parameter (such as serum high-density lipoprotein (HDL)) following bariatric surgery. The formula to compute BTI is as follows: BTI=(100×biochemical parameter prior to bariatric surgery−same biochemical parameter after surgery)/biochemical parameter prior to bariatric surgery). BTI is quite helpful to judge the surgical benefits and outcomes.

Statistical Analysis

The IBM SPSS Statistics for Windows, Version 26.0 (Released 2019; IBM Corp., Armonk, New York, United States) was used in analyzing the data. The data was expressed in tables and graphs using different forms of figures. The qualitative variables were expressed as numbers and percentages, and the quantitative data (descriptive data) was expressed as the mean and standard error of the mean. Paired sample t-test was used to compare the data before and after treatment. The results will be considered highly significant when the significant probability is less than 0.001 (***p-value <0.001). Data was collected, analyzed, and presented as the mean±standard error of the mean using the SPSS software. *** denotes that p<0.001 (utilized as a significance indicator).

## Results

A total of 61 patients were enrolled in this study. Regarding the demographic characteristics, the mean age of the studied participants was 35.639±7.882, and the age distribution was mostly in the range of 30-49 years, with 45.9% falling in the 30-39-year age group and 32.79% in the 40-50-year age group. The youngest age group (18-29 years) composed 21.31% of the participants in the study (Table 1).

**Table 1:** Basic characteristics of the studied patients (n=61) SD: standard deviation; BMI: body mass index

Characteristics	Category	Study group (n=61)	
		No.	%
Gender	Female	37	60.65
	Male	24	39.35
Age	18-29	13	21.31
	30-39	28	45.9
	40-50	20	32.79
Nationality	Saudi	51	83.6
	Non-Saudi	10	16.4
Marital status	Single	10	16.4
	Married	45	73.77
	Divorced	6	9.83
	Widowed	0	0
Employment	Yes	44	72.13
	No	17	27.87
Special habits	Smoking	15	24.59
	Alcohol	0	0
Age (years) (mean±SD)	35.639±7.882		
BMI (kg/m^2^) (mean±SD)	45.528±7.791		

**Figure 1:**
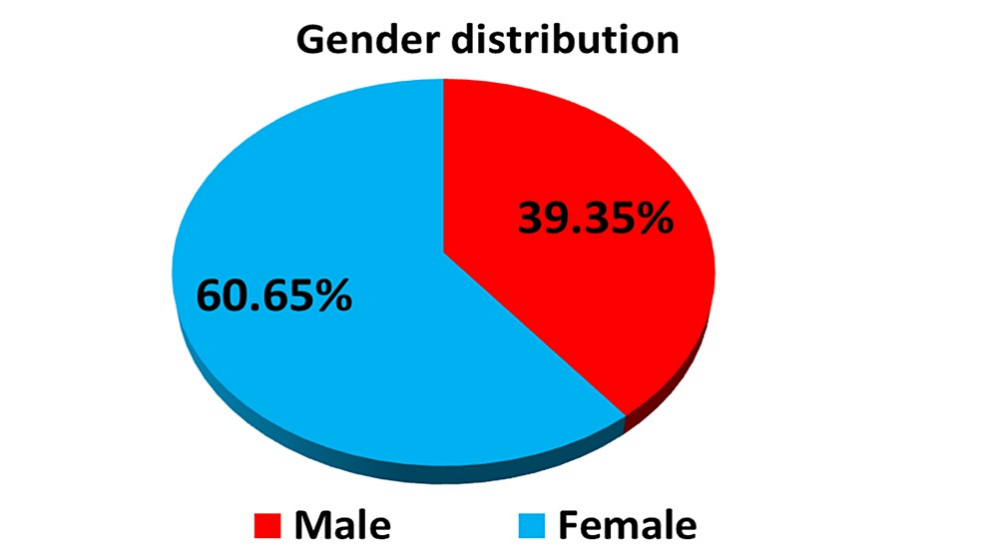
A pie chart showing the gender distribution among the studied groups (n=61)

**Figure 2:**
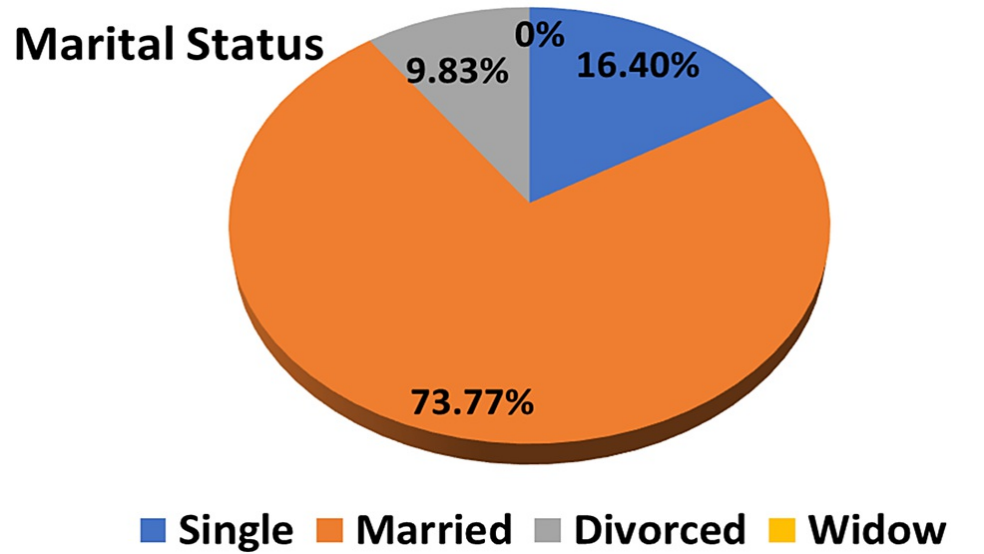
A pie chart showing the marital status distribution among the studied groups (n=61)

Gender distribution showed that most of the participants were females (60.65%) and 39.35% were males (Table 1 and Figure 1). The majority of participants were Saudis (83.6%) and 16.4% were non-Saudis (Table 1), while in terms of employment, about 72.13% were employed and 27.87% were non-employed (Table 1). Regarding marital status, most of them were married (73.77%), and only 9.83% (n=6) were divorced (Table 1 and Figure 2). 24.59% of the participants were smokers, and no one drank alcohol (Table 1).

**Figure 3:**
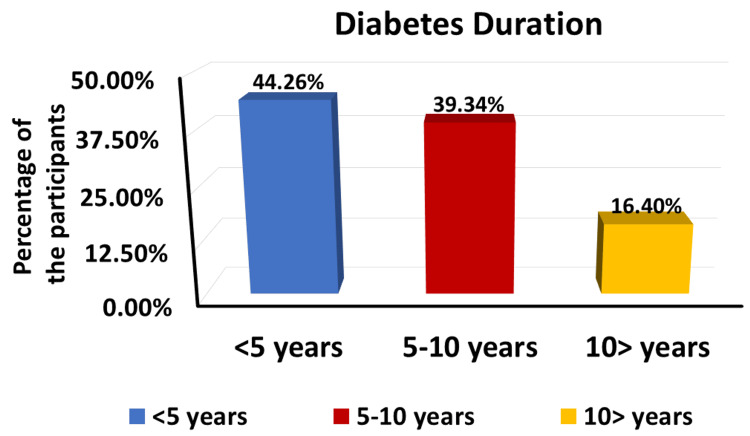
Diabetes duration among the studied groups (n=61)

**Figure 4:**
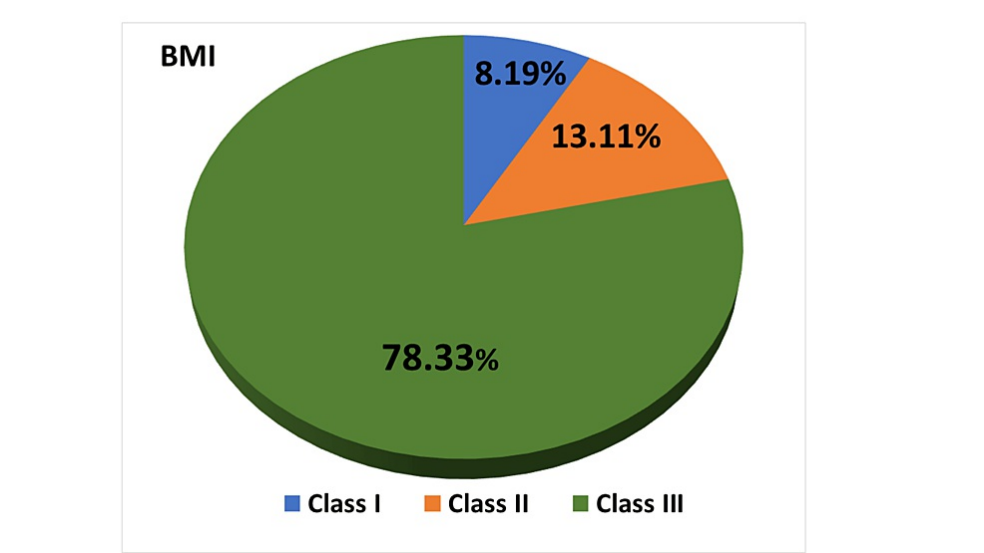
A pie chart showing the distribution of BMI among the studied groups (n=61) BMI: body mass index

In terms of diabetes duration, the largest group among the participants (44.26%) had diabetes for less than five years, followed by 39.34% who had diabetes for 5-10 years and 16.4% who had diabetes for more than 10 years (Figure 3). The BMI distribution showed that almost half of the participants (78.33%) had a BMI of 40 or greater (obesity class III), followed by 13.11% with a BMI between 35 and 39.9 (obesity class II) and 8.19% with a BMI between 30 and 34.9 (obesity class I). None of the participants had a BMI below 30 (non-obese) (Figure 4).

**Figure 5:**
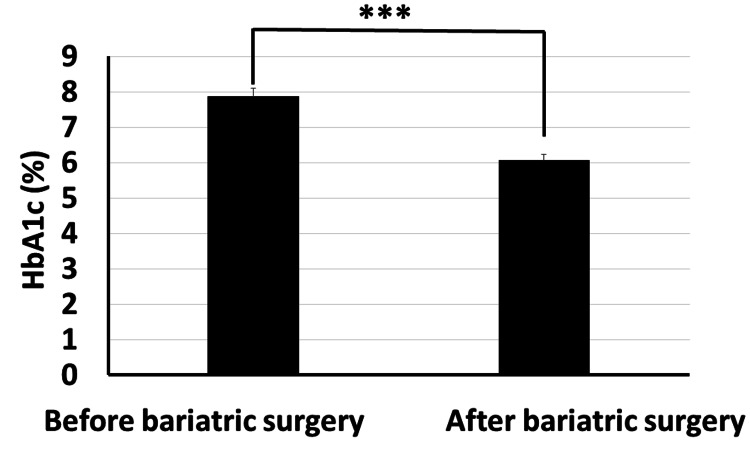
Bariatric surgery significantly decreased glycated hemoglobin in obese diabetic patients (p<0.001)

Regarding the metabolic parameters before and after surgery, they included glycemia (HbA1C) and lipidemia (lipid profile) parameters. The results showed statistically significant improvement in HbA1C in patients after surgery, as it significantly decreased from 7.874±1.824% before bariatric surgery to 6.061±1.350% after bariatric surgery with p<0.001 (Figure 5).

**Figure 6:**
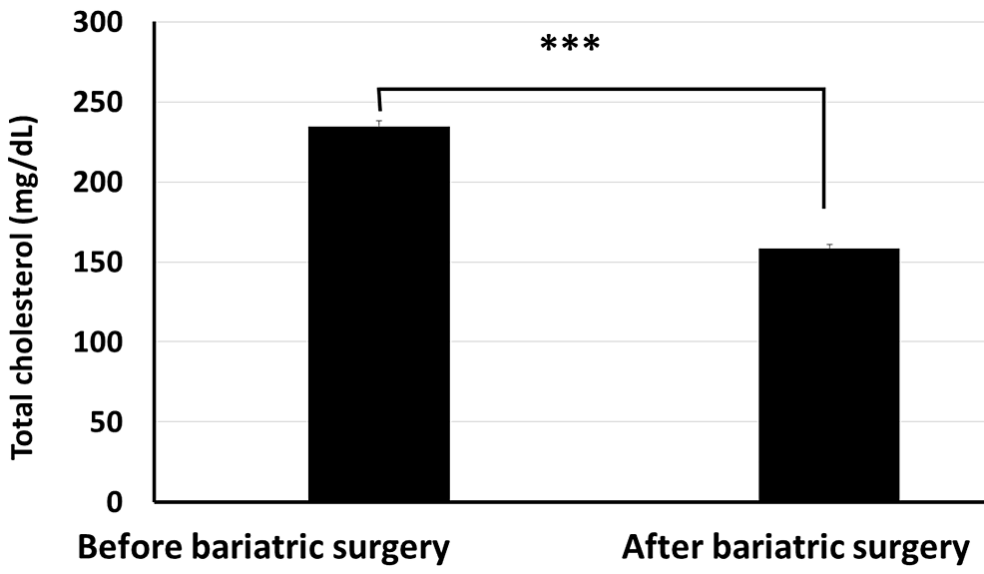
Bariatric surgery significantly decreased the serum total cholesterol in obese diabetic patients (p<0.001)

**Figure 7:**
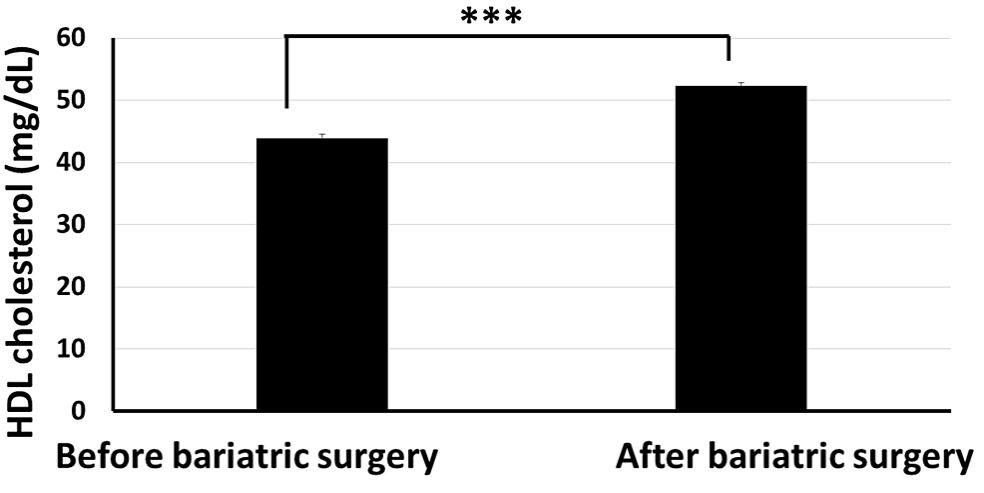
Bariatric surgery significantly increased the serum HDL cholesterol in obese diabetic patients (p<0.001) HDL: high-density lipoprotein

**Figure 8:**
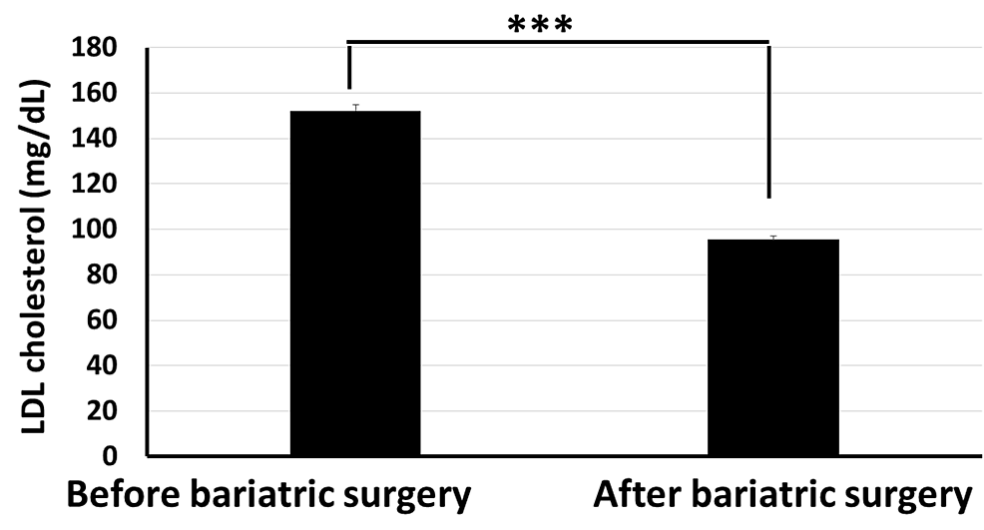
Bariatric surgery significantly decreased the serum LDL cholesterol in obese diabetic patients (p<0.001) LDL: low-density lipoprotein

**Figure 9:**
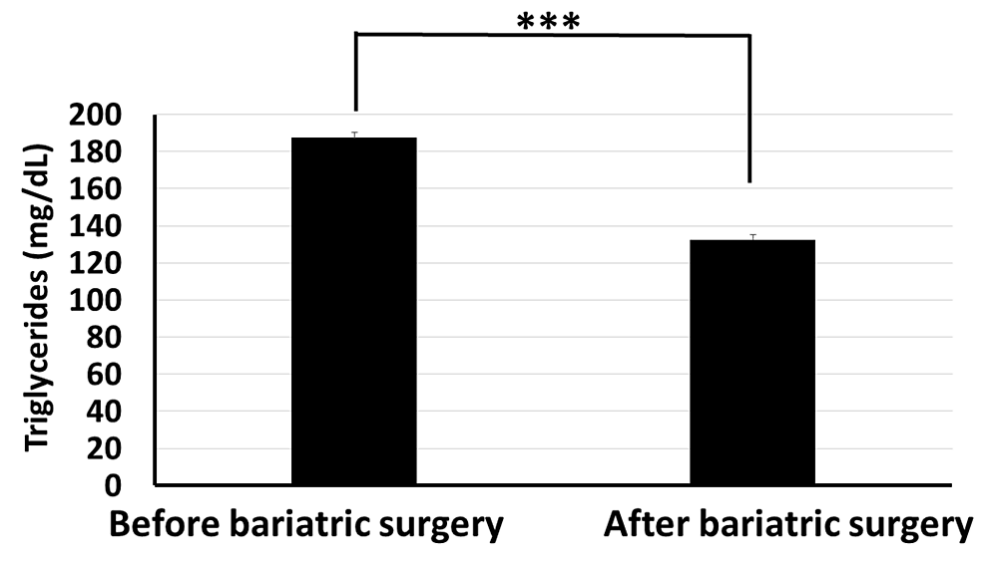
Bariatric surgery significantly decreased the serum triglycerides in obese diabetic patients (p<0.001)

**Figure 10:**
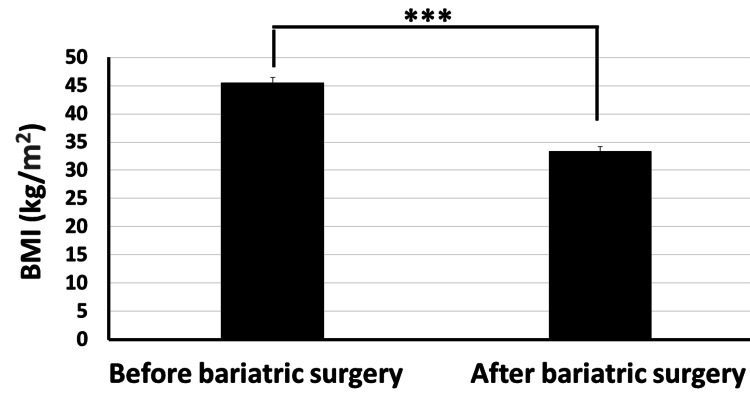
Bariatric surgery significantly decreased the BMI in obese diabetic patients (p<0.001) BMI: body mass index

Moreover, our data revealed that the total cholesterol level significantly decreased from 234.4±26.7 mg/dl before bariatric surgery to 158.4±17.3 mg/dl post surgery (Figure 6). The HDL cholesterol level increased significantly from 43.8±6.2 mg/dl before surgery to 52.3±4.6 mg/dl post surgery (p<0.001) (Figure 7). The LDL cholesterol level also showed a significant reduction from 152.2±19.4 mg/dl before surgery to 95.6±15.7 mg/dl post surgery (p<0.001) (Figure 8). Triglyceride levels also significantly decreased, from 187.3±24.6 mg/dl before surgery to 132.5±19.5 mg/dl post surgery (p<0.001) (Figure 9). In addition to these metabolic parameters, BMI showed a statistically significant decrease after surgery, as it decreased from 45.528±7.791 kg/m^2^ before surgery to 33.416±6.184 kg/m^2^ post surgery (p<0.000) (Figure 10).

BTI for parameters of both glycemia and lipidemia

As stated in the methodology section, BTI for the significant reductions is calculated as follows: BTI=(100×(biochemical parameter prior to bariatric surgery−same biochemical parameter after surgery)/biochemical parameter prior to bariatric surgery). BTI for HbA1C is calculated as 100×(7.87-6.06)/7.87=22.99%, i.e., glycated hemoglobin decreased by about 23% following bariatric surgery (Figure 5). BTI for serum total cholesterol is calculated as 100×(234.4-158.4)/234.4=32.42%, i.e., serum total cholesterol decreased by about 32.42% following bariatric surgery (Figure 6). BTI for serum HDL cholesterol is calculated as 100×(52.3-43.8)/43.8=19.4%, i.e., serum HDL cholesterol increased by about 19.4% following bariatric surgery (Figure 7). BTI for serum LDL cholesterol is calculated as 100×(152.2-95.6)/152.2=37.18%, i.e., serum LDL cholesterol decreased by about 37.18% following bariatric surgery (Figure 8). BTI for serum triglycerides is calculated as 100×(187.3-132.5)/187.3=29.26%, i.e., serum triglycerides decreased by about 29.26% following bariatric surgery (Figure 9).

CTI

As stated in the methodology section, CTI [12] is calculated as follows: CTI=(100×(the clinical parameter prior to bariatric surgery−same clinical parameter after surgery)/the clinical parameter prior to bariatric surgery). CTI for a reduction in BMI following bariatric surgery is calculated as 100×(45.52-33.41)=26.6%, i.e., BMI decreased by about 26.6% following bariatric surgery (Figure 10).

## Discussion

Lifestyle modifications, the use of medication, and using prophetic medicine remedies (alternate fasting, *Nigella sativa*, costus, natural honey, eating vinegar, and others) [13,14] are all effective ways to prevent and treat type 2 diabetes and other diseases including obesity, but it was shown that bariatric surgery may also reduce the risk of developing type 2 diabetes mellitus in obese people. The main findings of this study were promising in terms of the therapeutic benefits of bariatric surgery on the adequate control of the level of HbA1C and reducing the BMI among obese patients with type 2 diabetes mellitus in a Saudi tertiary health center (King Fahad General Hospital, Al-Madinah Al-Munawwarah, Saudi Arabia). Several types of bariatric surgeries were approved by the American Society for Metabolic and Bariatric Surgery (ASMBS) to maintain weight loss through various mechanisms [15]. Several clinical trials have revealed the efficacy of bariatric surgeries and their sustainability to maintain weight loss in addition to relieving many of the medical comorbidities associated with obesity when compared with other conventional methods [16].

The findings of this study have proved that bariatric surgery may be a vital treatment option for diabetic patients who are obese and who are unable to maintain adequate glycemic control with the use of more conventional techniques, as we observed statistically significant improvement in HbA1C in patients after surgery where it significantly decreased from 7.874±1.824 mg/dl before surgery to 6.061±1.350 mg/dl after surgery (p<0.001). Using the novel CTI, bariatric surgery decreased BMI by about 26.6%. Using the novel BTI, bariatric surgery decreased HbA1C, serum total cholesterol, serum LDL cholesterol, and serum triglycerides by about 22.99%, 32.42%, 37.18%, and 29.26%, respectively, while serum HDL increased by about 19.4%.

Our data is consistent with the report of Courcoulas et al. [17], who reported a 25% reduction in body weight and a remission of the use of antidiabetic drugs after two years of surgery and then no medication after three years. This glycemic control after surgery is attributed to an increased level of glucagon-like peptide-1, which has a role in stimulating insulin secretion and decreasing glucagon and gastric emptying [18]. Another randomized controlled trial in an Italian center showed diabetes remission after five years of follow-up among surgically treated patients compared to medically treated patients [19]. This is in agreement with other studies that reported the remission of type 2 diabetes mellitus and good glycemic control following both sleeve gastrectomy and Roux-en-Y gastric bypass (RYGB) [11,20].

The timing of the application of bariatric surgery during the course of diabetes, which has a benefit-risk ratio, is still debated; however, the International Diabetes Federation suggested the indication for bariatric surgery for severe and less obese patients with poorly controlled type 2 diabetes mellitus not controlled by lifestyle or medical management [21], while other studies reported the efficacy of bariatric surgery in diabetic remission and preventing the progression of micro- and macrovascular complications when the duration of diabetes is short [22]. Other long-term controlled Swedish studies reported a decrease in the incidence of type 2 diabetes mellitus in severely obese patients after bariatric surgery [23].

This explains the significant remission of diabetes among the studied participants, as the largest group (44.26%, n=27) had diabetes for less than five years, followed by 39.34% (n=24) who had diabetes for 5-10 years, which is consistent with the evidence of the application of surgery during the early course of the disease [24].

Regarding the metabolic parameters and adiposity before and after surgery, in this study, BMI showed a statistically significant decrease after surgery, as it decreased from 45.528±7.791 kg/m^2^ before surgery to 33.416±6.184 kg/m^2^ post surgery with a p-value of <0.001. Moreover, our results reported a statistically significant reduction in the total cholesterol level, LDL, and triglycerides, in addition to a statistically significant improvement in the HDL level.

Mazidi et al. [25] found a marked effect of bariatric surgery on adiposity and metabolic profile, which was detected by a reduction in BMI and body fat after one year postoperatively, a marked improvement in HDL, and a reduction in cardiovascular risk factors such as total cholesterol level, triglycerides, and LDL level. Other studies reported 20% body weight loss after two and six years postoperatively [26]. This is consistent with Khorgami et al. [27] and Ding et al. [28], who reported a favorable effect of bariatric surgery on the metabolic profile of patients compared to medical management; they proved to be the most effective alternative management of diabetic patients with cardiometabolic disorders. The exact mechanism of bariatric surgery for improving the metabolic profile is still elusive; however, the hypothesis that bariatric surgery increases the intestinal peptides, which play a role in the regulation of energy expenditure and appetite, is considered to be the main cause of weight loss [29]. A systematic review of findings had provided evidence for a substantial and significant improvement in physical and mental health favoring the surgical group compared with controls spanning 5-25 years after surgery [30].

The outcome of this study indicates that bariatric surgery may be a vital therapeutic option for obese diabetic patients who are having difficulty achieving glycemic control with the use of standard techniques. Strength points in this study included the relatively young ages of the investigated sample (<50 years), the introduction of the novel indices (CTI and BTI), and the data on lipid profiles and glycemia in addition to the geographically studied region (western Saudi Arabia). We have to consider some limitations of this study, namely, small sample size, retrospective study design, absence of a control group, and short duration of follow-up, so we cannot correlate the short- and long-term overall effects of bariatric surgeries.

For the authors, we recommend bariatric surgery for treating obese patients who have type 2 diabetes and consider it an alternative therapy for the management of cardiometabolic conditions. We do recommend bariatric surgery for treating obesity and obesity-related metabolic disturbances in Saudi Arabia as insulin-resistant diabetes mellitus.

The recommendations in our study are promising. The prevention of complications seen in obese diabetic patients can be reduced by losing weight through various weight loss models. It is necessary to have randomized cohort studies for the follow-up of obese patients with comorbidities such as diabetes mellitus and cardiovascular diseases, as most of the studies are observational. More studies are needed for long-term follow-up for the detection of the complications of bariatric surgeries regarding nutritional deficiencies and quality of life.

## Conclusions

Following bariatric surgery, there were maximal and significant decreases (p<0.001) in BMI and HbA1C, serum triglycerides, serum LDL cholesterol, and serum total cholesterol. It's interesting to note that bariatric surgery significantly increased serum HDL (p<0.001). As bariatric surgery leads to weight loss, improved diabetes control, and an improvement in the metabolic profile, it is regarded as a successful intervention for obese diabetic patients. Given the high rates of diabetes and obesity in Saudi Arabia, surgery may be advised. Our data suggests that bariatric surgeries can achieve better HbA1C control and diabetic remission in obese patients who are having trouble achieving glycemic control through the use of traditional methods. Bariatric surgery is becoming an increasingly essential therapy option for obese patients who have type 2 diabetes and consider it an alternative therapy for the management of cardiometabolic conditions.
